# Coexisting Lung Cancer and Pulmonary Tuberculosis: A Comprehensive Review From Incidence to Management

**DOI:** 10.1002/cnr2.70213

**Published:** 2025-05-10

**Authors:** Wendi Zhou, Hongxu Lu, Jiamin Lin, Jialou Zhu, Jizhen Liang, Yalin Xie, Jinxing Hu, Ning Su

**Affiliations:** ^1^ State Key Laboratory of Respiratory Disease, Guangzhou Key Laboratory of Tuberculosis Research, Guangzhou Chest Hospital, Institute of Tuberculosis Guangzhou Medical University Guangzhou P. R. China; ^2^ Department of Children's Psychological and Rehabilitation, Shen Zhen Maternity and Child Health Hospital Southern Medical University Shenzhen P. R. China; ^3^ Department of Oncology Guangzhou Red Cross Hospital Guangzhou P. R. China

**Keywords:** chronic inflammation, clinical management, DNA damage, immune checkpoint inhibitors, lung cancer, tuberculosis

## Abstract

**Background:**

Globally, infections account for 10% of new cancer cases, and cancer can compromise the immune system, increasing the risk of infections. With advances in cancer treatment, widespread use of immunotherapy, and prolonged survival of cancer patients, the coexistence of tuberculosis (TB) and cancer is becoming increasingly common in clinical settings.

**Aim:**

This review aims to explore the interaction between tuberculosis (TB) and tumors, particularly lung cancer (LC), and to identify appropriate clinical management approaches.

**Results:**

LC patients with a history of TB have higher adjusted risk ratios for both all‐cause and cancer‐specific 3‐year mortality compared to those without a history of TB. TB may elevate the risk of developing tumors through mechanisms such as chronic inflammation, altered immune responses, and DNA damage. Conversely, cancer patients, whether due to the disease itself or immune dysfunction caused by anti‐tumor treatments, may be more susceptible to TB. The coexistence of TB and tumors presents significant challenges in clinical management, making the development of treatment strategies and quality‐of‐life improvements crucial.

**Conclusion:**

There is a close relationship between TB and cancer, with TB potentially serving as a risk factor for cancer, and cancer influencing susceptibility to TB. Effective clinical management is essential to enhance treatment strategies and improve the quality of life for patients with both TB and cancer.

## 1 Introduction

Tuberculosis (TB), a chronic infectious disease caused by *Mycobacterium tuberculosis (Mtb)*, is the second deadliest infectious disease after COVID‐19. China ranks third among the 30 high‐burden countries, accounting for 7.4% of the global TB cases [1]. An estimated quarter of the world's population is infected with *Mtb*, with approximately 5%–10% of TB infections progressing to active TB [2]. TB remains a major global health issue, especially multi‐drug resistant tuberculosis (MDR‐TB), with treatment success rates typically below 60%. The risk of potential TB infection due to MDR strains is twice as high in children as that in adults [3, 4]. China has the second highest burden of MDR‐TB, presenting an urgent challenge in controlling its spread [5, 6].

In 2020, cancer was the second leading cause of death globally, resulting in nearly 10 million deaths (approximately one‐sixth of all deaths) [7]. Lung cancer (LC) is the leading cause of cancer‐related death worldwide. In 2022, China reported approximately 871 000 new LC cases and 767 000 new LC deaths, ranking first among malignant tumors in the country [8]. LC is a heterogeneous disease primarily classified into two subtypes: small‐cell lung cancer (SCLC) and non‐small‐cell lung cancer (NSCLC) [9]. As of 2023, the 5‐year survival rate for SCLC is 12%, while NSCLC has a relatively higher 5‐year survival rate of approximately 22% [10].

Worldwide, 10% of new cancer cases are attributed to infections, and cancer can affect the immune and other systems, increasing the risk of infections [11]. With advancements in cancer treatment, widespread application of immunotherapy, and prolonged survival of cancer patients, the coexistence of TB and cancer is becoming increasingly common in clinical settings [12]. In the case of LC, studies have categorized coexistence into three causes: TB increasing LC risk, LC leading to TB reactivation, and coincidental coexistence of two common diseases [13]. Previous studies have indicated that pulmonary tuberculosis (PTB) is associated with an increased risk of cancer incidence [14, 15, 16, 17, 18, 19, 20], mortality, and poor prognosis [21, 22]. Meanwhile, related literature suggests an increased risk of TB in cancer patients [23, 24, 25, 26, 27]. TB may be a risk factor for cancer development, primarily through chronic inflammation and immune dysfunction. At the same time, cancer may increase individual susceptibility to TB.

While existing literature primarily focuses on individual aspects of TB and cancer—such as epidemiology, clinical management, and molecular mechanisms—the interaction between these two diseases, especially the immune and molecular pathways that drive their coexistence, remains insufficiently explored. This study aims to address this gap by providing a comprehensive review of the bidirectional relationship between TB and cancer. Specifically, we will examine how TB may contribute to cancer development and how cancer, in turn, heightens susceptibility to TB. By integrating these perspectives, this review offers new insights that will enhance our understanding of the complex interplay between these diseases and its implications for clinical practice and future research.

## 2 Cancer Risk in Tuberculosis Patients: Epidemiology and Mechanisms

### 2.1 Epidemiology of Cancer in Tuberculosis Patients

Numerous epidemiological studies have shown that TB patients face a higher risk of cancer development, along with increased mortality and poorer survival outcomes [17] (Table 1). Specifically, there is a notable association between PTB and LC, with preexisting active PTB increasing the risk of LC. Liao et al. revealed that LC patients with a history of TB have increased adjusted risk ratios for all‐cause and cancer‐specific 3‐year mortality, compared to those without TB history [22]. This finding underscores that a TB history not only raises the risk of LC but also impacts the long‐term prognosis of LC patients [21]. In China, the incidence of lung cancer in patients with pulmonary tuberculosis was 2.6%, and it was 9.95% in the elderly [28]. Further studies considering factors such as age and sex found that younger PTB patients are more likely to develop LC than older PTB patients, and female PTB patients have a higher risk of dying from LC than males [18, 29]. Therefore, regular chest X‐ray or chest CT examinations for young female patients with PTB to prevent LC are particularly important.

**TABLE 1:** Epidemiological correlation study data between tuberculosis and lung cancer.

Author (year)	Country/Region	Study population	Methods	Results (HR/OR.etc)	References
Zheng (1987)	Shanghai, China	2900 patients	Case–control study	Adenocarcinoma, OR = 3.2, 95% CI [1.9–5.5]; Squamous, OR = 2.6, 95% CI [1.5–4.6]	[19]
Yu (2011)	Taiwan, China	716 872 subjects	Retrospective cohort study	HR = 4.37 95% CI [3.56–5.36]	[16]
Heuvers (2011)	Rotterdam	7983 study participants	Prospective cohort study	HR = 2.36 95% CI [1.1–4.9]; shorter survival than those without a history of PTB, with a mean difference of 311 days	[21]
Wu (2011)	Taiwan, China	82 435 patients	Retrospective cohort study	Respiratory: HR = 1.67 95% CI [1.42–1.96] Digestive tract: HR = 3.09, 95% CI [2.42–3.94] Hematologic: HR = 3.22 95% CI [1.98–5.22]	[23]
Lai (2017)	Taiwan, China	2522 patients	Retrospective cohort study	HR = 2.90 95% CI [2.11–3.99]	[27]
Dobler (2017)	—	921 464 patients	Meta‐analysis	Adult cancer: IRR = 2.61 95% CI [2.12–3.22] Hematologic malignancies: IRR = 3.53 95% CI [1.63–7.64] Adult solid tumors: IRR = 2.25 95% CI [1.96–2.58]	[25]
Shu (2019)	Taiwan, China	1 185 221 patients	Retrospective cohort study	Respiratory: SIR = 5.45 95% CI [4.92–5.97] Hematology: SIR = 3.70 95% CI [3.46–3.93] Head and neck: SIR = 2.58 95% CI [2.37–2.80]	[26]
Leung (2020)	195 countries	52 480 cancer cases	Meta‐analysis	RR = 1.69 95% CI [1.46–1.95]	[14]
Chen (2021)	Xinjiang, China	45 455 patients	Case–control study	OR = 1.68 95% CI [1.43–1.97]	[15]
Abdeahad (2022)	—	4751 patients	Meta‐analysis	RR = 2.170 95% CI [1.833–2.569] *p* < 0.001	[17]
Oh (2022)	Korean	20 252 participants	Retrospective cohort study	HR = 3.24 95% CI [1.87–5.62]	[18]
Cabrera‐Sanchez (2022)	Latin American and Caribbean	6240 records	Meta‐analysis	HR = 5.01 95% CI [3.64–6.89]	[20]
Liao (2023)	Taiwan, China	43472 patients	Retrospective cohort study	3 year all‐cause mortality HR = 1.13 95% CI [1.04–1.23] 3 years cancer‐specific mortality: HR 1.11 95% CI [1.02–1.21]	[22]
Liao (2023)	Taiwan, China	1335 patients	Retrospective cohort study	Age: 60–69 years, HR = 1.4 95% CI [1.1–1.8] ≥ 70 years, HR = 1.9 95% CI [1.5–2.4] Gender: Male HR = 1.7 95% CI [1.5–2.0]	[24]

Abbreviations: HR, hazard ratio; IRR, incidence rate ratio; RR, relative risk; SIR, standardized incidence rate.

### 2.2 Mechanisms Through Which Tuberculosis Contributes to Cancer Development

The mechanisms by which TB induces cancer are multifaceted and involve chronic inflammation, altered immune responses, DNA damage, tissue remodeling, changes in gene expression, and regulation of the tumor microenvironment. These mechanisms promote cancer development and progression.

#### 2.2.1 Role of Reactive Oxygen Species in *Mtb*‐Induced LC

*Mtb* infection triggers host cell responses and significantly increases reactive oxygen species (ROS) and nitric oxide levels in macrophages and alveolar epithelial cells [30, 31, 32, 33]. Mitochondrial ROS play a crucial role in this process by damaging mitochondrial membranes and exacerbating lung inflammation, and fibrosis [34]. These changes involve ROS‐induced damage to chromosomal integrity and mitochondrial DNA, which leads to lung tissue reconstruction and dysfunction. ROS play a key role in LC development, with excessive ROS causing DNA damage, producing mutagenic mediators such as peroxynitrite and increasing cellular genetic instability [35, 36]. Additionally, increased ROS levels may be associated with idiopathic pulmonary fibrosis by stimulating the release of danger‐associated molecular patterns [37]. Mitochondrial DNA damage also leads to dysfunction in the electron transport chain, resulting in mitochondrial dysfunction, apoptosis of alveolar epithelial cells, and development of pulmonary fibrosis, providing a biological basis for LC formation [38, 39]. Studies have also shown that ROS can upregulate the expression of oncogenes Jun and Fos, affecting cell proliferation and cycle regulation. Activation of Jun/Fos leads to the inhibition of P21 expression, causing cell cycle alterations, accelerating cell division rates, and shortening DNA repair time, thereby significantly increasing the likelihood of cellular carcinogenesis [40, 41, 42, 43]. Researchers are exploring methods to reduce LC risk by lowering ROS levels or improving *Mtb* infection treatments, including the use of antioxidants and the development of more effective anti‐TB drugs or vaccines. However, the current research still faces limitations in sample size, long‐term follow‐up, and multivariable analysis. Future research needs to consider how genetic background, lifestyle, and environmental factors interact with *Mtb* infection and affect LC risk.

#### 2.2.2 Chronic Inflammation From *Mtb* Infection and Its Role in Cancer Development

Chronic inflammation induced by *Mtb* infection is crucial for LC development. *Mtb* infection typically leads to the formation of granulomas in the lungs, with the center containing mycobacteria, surrounded by myeloid and lymphoid cells. Granuloma characteristics, such as caseous necrosis, cavity formation, and fibrosis, can lead to lung scarring. Approximately 20% of PTB patients who receive initial treatment develop lung scarring, with scar tissue accounting for 30%–57% of lung scar cancer cases [44]. Inflammatory cytokines produced during this process may lead to an imbalance in cell proliferation and apoptosis, thereby promoting tumor development.

Research on the relationship between *Mtb* infection and LC risk dates back to the early 20th century, initially focusing on pathology, and recently delving into molecular and cellular biology. Many epidemiological studies have shown a correlation between *Mtb* infection and LC incidence, but the specific causal relationship remains to be explored. T cells and macrophages play key roles during Mtb infection. T cells produce interferon‐γ (IFN‐γ) to respond to *Mtb* infection, whereas activated macrophages release inflammatory cytokines, ROS, prostaglandins, and proteases to eliminate bacteria [45]. Although this complex immune response helps to control *Mtb*, it may also lead to lung tissue damage. Repeated tissue necrosis, regeneration, and damage episodes may cause disordered cell proliferation and angiogenesis, increasing LC risk [46, 47].

Tumor development is a complex process involving changes in the tumor microenvironment, in which chronic inflammation creates conditions conducive to tumor progression [48]. Inflammatory cytokines, such as INF‐γ, interleukin‐1 (IL‐1), IL‐2, IL‐12, and tumor necrosis factor (TNF), secreted during *Mtb* infection promote lung tissue inflammation [49]. The balance between cell proliferation and apoptosis is crucial for tumor development. Inflammatory factors, such as Toll‐like receptor‐2 (TLR‐2), IL‐6, IL‐17, and IL‐22, which are highly expressed in the serum of patients with TB and LC, are closely related to this process. Studies have shown that silencing TLR2 promotes apoptosis, thereby affecting tumor development [50]. These findings highlight the key role of inflammatory factors in regulating the tumor microenvironment and cell fate. Bours et al. pointed out that nuclear factor (NF) and IL‐6 might upregulate anti‐apoptotic genes in the NF‐κB pathway, influencing tumor cell survival [51]. The NF‐κB pathway plays an active role in LC [52] and other respiratory diseases, as well as in microbial infections, including TB [53, 54]. These findings underscore the dual role of NF‐κB signaling in TB and LC pathogenesis and the potential bridging role between these two diseases. Thus, *Mtb* may promote tumor development by activating inflammatory factors. Research has shown that chronic *Mtb* infection may promote the development of lung squamous cell carcinoma, with correlations observed in mouse models and TB patients [48]. *Mtb* infection leads to the proliferation and damage of lung epithelial cells [55, 56] while activating macrophages, dendritic cells, and type II alveolar cells to release pro‐inflammatory and anti‐inflammatory cytokines (such as IL‐1, IL‐6, IL‐17, IL‐18, IL‐22, TNF‐α, IFN‐γ, and IL‐10), further promoting lung epithelial cell proliferation [57].

Interventions targeting these cytokines and signaling pathways, such as inhibition of the NF‐κB pathway, may help prevent LC caused by TB infection. Recent studies suggest that in *Mtb*‐infected macrophages, the prostaglandin E2/cyclooxygenase 2 (COX‐2) signaling pathway is activated, possibly leading to immune response dysfunction that creates a microenvironment conducive to *Mtb* survival and replication [58]. *Mtb* infection induces COX‐2 upregulation in dendritic cells and monocytes, promoting tumor metastasis through the p‐Akt‐NF‐κB pathway that affects matrix metalloproteinase‐9 activity [59]. In addition to affecting tumor metastasis, COX‐2‐produced prostaglandin E2 mediates apoptosis inhibition and BCL‐2 synthesis enhancement [60, 61], thereby increasing the risk of DNA damage and cancer.

Long‐term *Mtb* infection leads to sustained T cell activation followed by functional failure, which manifests as decreased cytokine production, diminished proliferation, and reduced killing function. T‐cell depletion reduces the ability of the immune system to control infection effectively. In addition, persistent *Mtb* infection promotes the expression of various immune checkpoint proteins on the surface of T cells, such as Programmed Cell Death protein‐1 (PD‐1), cytotoxic T lymphocyte‐associated protein‐4, and mucin domain‐containing protein 3 [62]. The upregulation of these proteins further weakens the immune system's response to *Mtb* infection [63]. The PD‐1/PD‐L1 signaling pathway can affect innate and adaptive immunity against *Mtb* infection to varying degrees. *Mtb* infection also leads to the upregulation of PD‐1, PD‐L1, and PD‐L2, reduces the production of IFN‐γ, and reduces the toxicity of CD8+ T cells [64]. In addition, studies have shown that PD‐1 knockout mice show more severe TB symptoms than wild‐type mice in the *Mtb* infection model, accompanied by a significant increase in proinflammatory cytokines, such as TNF and IL‐1 [65]. This suggests that PD‐1 plays an important role in the regulation of immune responses and inflammation. In addition, the overproduction of IFN‐γ may cause death in PD‐1 knockout mice, highlighting the complex interactions and balance between different components of the immune system [66]. In *Mtb* infection studies, mucin domain‐containing protein 3 was found to interact with its ligand, galectin‐9, in *Mtb*‐infected macrophages. This interaction induces IL‐1β production and inhibits *Mtb* growth [67], revealing a critical role of mucin domain‐containing protein 3 in regulating macrophage responses to *Mtb* infection. In the late stage of chronic *Mtb* infection, mucin domain‐containing protein 3 and PD‐1 positive CD4+ and CD8+ T cells co‐express other T cell immunoglobulin domains. These cells produce more anti‐inflammatory cytokines, such as IL‐10, while reducing the production of pro‐inflammatory cytokines such as IFN‐γ, TNF‐α, and IL‐2, and show features of functional exhaustion [68]. This phenomenon is closely related to the poor prognosis of various cancers, indicating the important role of immune checkpoints in tumor immune escape and cancer progression.

#### 2.2.3 *Mtb*‐Induced EGFR Mutations and Epiregulin Production in LC

Epidermal growth factor receptor (EGFR) is a transmembrane protein involved in various biological processes, including cell proliferation, apoptosis, and survival [69]. EGFR activation regulates tumor cell behavior through downstream signaling pathways such as extracellular signal‐regulated kinase and p38 MAPK [70]. These pathways regulate cell proliferation, differentiation, migration, and survival. Abnormal EGFR activation or mutation can lead to sustained activation of these signaling pathways, promoting tumor development and progression. The overall EGFR mutation rate in Asian patients with lung adenocarcinoma is 51.4%, with a rate of 50.2% in China [71]. A study in Korea found that 183 (39%) of 477 lung adenocarcinoma patients had EGFR mutations, and 100 (21%) had TB lesions. The EGFR mutation frequency was significantly higher in the TB group than in the non‐TB group (*p* < 0.05), and TB‐associated LC patients receiving EGFR‐TKIs treatment had poorer treatment response and survival rates (*p* < 0.05) [72].

*Mtb* infection affects LC development through complex molecular mechanisms. Experimental studies [48] have shown that *Mtb*‐infected macrophages can induce DNA damage and produce epiregulin, which may act as an extracellular survival and growth factor promoting tumor development, especially in squamous metaplasia and tumor formation. Moreover, epiregulin expression is associated with lymph node metastasis and shortened survival in NSCLC, and the invasiveness of NSCLC cell lines with activating EGFR mutations can be reduced by silencing epiregulin or by using antibody treatments [73, 74]. Thus, *Mtb*‐induced EGFR mutations and epiregulin production may play an important role in LC development.

#### 2.2.4 Role of *Mtb* Protein Tyrosine Phosphatase in LC Development

*Mtb* can secrete various effector proteins into host cells, interfering with host cell signaling pathways and biological functions, promoting pathogen survival in host cells, and ultimately leading to host cell pathology [75]. *Mtb* effector protein tyrosine phosphatase (PtpA) can be secreted into host cells, bind to ubiquitin molecules, and be activated by them, thereby dephosphorylating host p‐JNK and p‐p38, inhibiting JNK/p38 signaling pathway activation, and suppressing host immune function. PtpA can also competitively bind to the ubiquitin‐interacting domain of the host adapter protein TAB3, blocking the ubiquitin chain‐mediated transmission of NF‐κB signaling by TAB3, and inhibiting innate immunity [76].

Further research revealed that the PtpA host interaction protein TRIM27 (a ubiquitin ligase) can act as a host restriction factor, inhibiting *Mtb* survival in macrophages. However, PtpA can antagonize TRIM27‐mediated antibacterial immunity by binding to the RING domain of the TRIM27 protein [77]. Recent studies indicate [55] that *the Mtb* effector protein PtpA can regulate host cell immunity and cell behavior, affecting immune signals in the cytoplasm and entering the nucleus to inhibit specific genes such as GADD45A, promoting lung cancer cell proliferation and migration. This effect also enhances tumorigenicity in nude mice, primarily through PtpA DNA binding rather than through phosphatase activity. *Mtb* affects host cell signaling and immune function through the effector protein PtpA, potentially promoting tumor development.

## 3 Tuberculosis Risk in Cancer Patients: Epidemiology and Mechanisms

### 3.1 Epidemiology of Tuberculosis in Cancer Patients

Globally, cancer is considered an independent risk factor for TB, particularly in China, India, and Indonesia, with respiratory system malignancies being the most common [78]. Studies have found that cancer patients receiving immune checkpoint inhibitors (ICIs) have an eight‐fold higher incidence of TB than ordinary patients [38]. Various studies have reported a higher risk of TB in patients with different cancer types and stages (Table 1). A study in Taiwan noted that patients with LC undergoing surgery and chemotherapy had a significantly increased TB risk [24]. Patients with head and neck cancer have a three‐fold higher TB risk than the general population [27].

### 3.2 Tuberculosis Susceptibility in Lung Cancer Patients

#### 3.2.1 Role of Tumor‐Associated Macrophages in *Mtb* Susceptibility in LC

The immune system plays a critical role in lung cancer development and involves macrophages, dendritic cells, NK cells, and T cells. Tumor‐associated macrophages (TAMs) exert immunosuppressive effects in the LC immune microenvironment. Once LC patients are infected with *Mtb*, TAMs on the surface of PD‐L1 bind to inhibitory receptors on T cells, leading to effector T cell exhaustion and production of immunosuppressive factors such as IL‐10, TGF‐β, and CXCL8, reducing CD8+ T cell infiltration, thus inhibiting the immune response to *Mtb* and the adaptive immune response [79, 80]. Additionally, *Mtb* can prevent lysosome and phagosome fusion in macrophages, allowing them to survive and replicate in macrophages, ultimately leading to active TB in LC patients [81]. NK cells, as the first line of defense for monitoring and clearing tumor cells, often experience dysfunction or frequency reduction in LC patients, inhibiting NK cell toxicity toward infections and increasing susceptibility to active TB in LC patients [82].

#### 3.2.2 Impact of Antitumor Treatments on Tuberculosis Susceptibility in Lung Cancer Patients

From another perspective, taking LC as an example, antitumor treatment may increase the risk of developing active TB, with immunotherapy, chemotherapy, radiotherapy, and targeted therapy potentially increasing TB susceptibility or causing reactivation.

Immunotherapy has become increasingly important in the treatment of advanced LC and NSCLC, as it significantly prolongs the overall survival of patients. Immunotherapy has become the standard first‐ and second‐line treatment for advanced lung cancer. However, ICI treatment may lead to immune‐related adverse events, with incidence rates ranging from 54% to 76% [83]. Recent studies have indicated that LC patients receiving ICI treatment may experience TB reactivation or rapid TB progression. A systematic comprehensive description identified that PD‐1 and PD‐L1 blocking immunotherapy in cancer patients increased the risk of TB reactivation, with an estimated TB incidence rate 35 times higher than that in the general population and a high mortality rate of approximately 22.2% [84]. Zaemes et al. assessed the association between ICI use and TB development, reporting 16 LC patients developing active TB during PD‐(L)1 inhibitor treatment, with a median time to TB recurrence of 6.3 months after starting ICI treatment [28].

#### 3.2.3 Mechanisms of Tuberculosis Reactivation During Cancer Immunotherapy

Based on this phenomenon, two potential mechanisms for TB reactivation in the context of cancer immunotherapy have been summarized in the existing literature: (1) immune suppression‐induced immune therapy infection, where the balance between the host immune response and pathogen invasion affects disease outcome and progression after *Mtb* infection. Day et al. described [85] the relationship between *Mtb*‐specific CD4+ T cell PD‐1 expression and human TB bacterial load, using peripheral blood samples from LTBI and TB patients, and found significantly higher PD‐1 expression levels on Th1 + CD4 T cells in smear‐positive TB patients compared to smear‐negative TB and LTBI patients, with levels decreasing after completing anti‐TB treatment. These data suggested a close association between *Mtb*‐specific CD4+ T cell PD‐1 expression and *Mtb* infection. Therefore, Th1+ CD4T cell function suppression by ICIs provides favorable conditions for LTBI reactivation. (2) Immune disorder‐induced immune therapy infection, differing from the previous TB reactivation mechanism, is due to excessive inflammation caused by immune checkpoint inhibition, leading to different infection reactivation patterns. Research has shown that PD‐1/PD‐L1 axis disruption leads to an excessive inflammatory state conducive to mycobacterial growth. Studies in transgenic mice and monkeys have found that PD‐1 gene deletion or anti‐PD‐1 antibody treatment increased susceptibility to *Mtb*, leading to decreased survival and increased bacterial load [86, 87]. Additionally, studies using human three‐dimensional cell culture models have indicated that PD‐1 inhibition may accelerate *Mtb* growth through excessive TNF‐α secretion, activating *Mtb* infection [88].

These studies suggest that PD‐1 blockade negatively impacts host‐*Mtb* interactions even without any immune suppression, favoring pathogen proliferation rather than host control. In‐depth research on these mechanisms is important for preventing and managing the risk of TB reactivation during ICIs treatment.

#### 3.2.4 Radiotherapy and Tuberculosis Susceptibility in Lung Cancer

Radiotherapy is one of the traditional methods of cancer treatment, acting on tumor cells through ionizing radiation, damaging cell DNA, causing DNA single‐ or double‐strand breaks and cell apoptosis, and achieving therapeutic purposes. Studies have shown that radiotherapy affects the patient's immune system by altering the number, balance, and interaction of immune cells, inducing tumor immunity, participating in immunosuppression, or promoting anti‐tumor effects [89, 90]. Choi et al. found that [91] this infection might be related to radiation‐induced damage, as radiotherapy may promote immunosuppression by polarizing immunogenic macrophages to immunosuppressive phenotypes and inducing lymphocyte apoptosis. TB occurrence results from host and *Mtb* interactions, with opportunistic infections occurring when “the enemy” is strong and “we” are weak.

#### 3.2.5 Chemotherapy and Tuberculosis Risk in Cancer Patients

Traditional chemotherapy drugs inhibit cancer cell growth through various mechanisms, including inducing DNA breaks, interfering with DNA and RNA synthesis, affecting mitosis, and producing ROS by damaging mitochondria and slowing, stopping, or killing cancer cell growth [92]. However, these drugs may also have side effects on normal immune cells, leading to suppressed host immune responses [93]. A Saudi Arabian study on 203 cancer patients found that [94] approximately 13% of patients receiving chemotherapy had *Mtb* infection, with 3.9% having active TB, particularly in elderly patients with solid tumors. This infection mechanism may result from the inherent immunosuppressive properties of cancer, immune system suppression due to chemotherapy, and other host factors, collectively increasing patient susceptibility to TB and cancer, and weakening local infection defense mechanisms, making it difficult to effectively clear infections [95]. This finding suggests that targeted screening plans are necessary to prevent the coexistence of TB and cancer in cancer patients with a high TB activation risk.

#### 3.2.6 Targeted Therapy and Tuberculosis Reactivation in Lung Cancer

Targeted therapy, an important biological therapy, provides a more precise and effective cancer treatment approach by targeting molecular targets driven by positive genes. For example, in lung cancer, common targets include EGFR mutations, ALK gene rearrangements, and other oncogene‐driven changes, such as ROS1, BRAF, NTRK, MET, and KRAS [96]. These targeted therapies can act directly on tumor cells and induce immune changes in the tumor microenvironment. In lung adenocarcinoma patients with EGFR mutations or ALK rearrangements, first‐line targeted therapy is a routine treatment. For instance, EGFR mutations are very common in lung adenocarcinoma, with significant differences in prevalence in different regions, such as approximately 15% in Europe and up to 62% in Asia [71, 97]. Common targeted drugs such as cetuximab mediate antibody‐dependent cell‐mediated cytotoxicity in tumors, activate adaptive and innate immune responses, and bind to CD16 receptors on EGFR, NK cells, and dendritic cells, causing immune stimulation, potentially leading to immunosuppression [93, 98]. Reports of PTB induced by targeted therapy are limited. Existing case studies [99, 100] indicate that EGFR‐TKI‐treated EGFR‐mutant NSCLC patients may develop PTB or PTB reactivation, possibly related to non‐TB lesion area *Mtb* reactivation caused by LC or increased opportunistic infection risk due to tumor treatment‐induced immunosuppression.

## 4 Clinical Management of Cancer Patients With Tuberculosis

Treatment of TB combined with cancer often faces the following challenges: treatment sequence, drug interactions, adverse reactions, and prognosis. Treatment plans vary for different cancer stages. For example, in the case of LC, early‐stage LC‐PTB is treated surgically, whereas late‐stage LC‐PTB is primarily treated with chemotherapy, immunotherapy, and targeted therapy.

During the surgical treatment of early stage LC‐PTB, issues such as whether anti‐TB treatment increases surgical risk, the optimal duration of preoperative anti‐TB treatment, and postoperative anti‐TB treatment duration need to be addressed. Clinical evidence suggests that using a strengthened four‐drug regimen for 2 weeks in drug‐sensitive TB patients can significantly reduce active *Mtb* in sputum [101, 102]. Sputum smear conversion time may be extended by a high initial sputum bacterial load and cavitary lesions. The limited literature on the surgical treatment of LC‐PTB suggests that tumor resection surgery after 2–3 weeks of anti‐TB treatment or simultaneous anti‐TB treatment and adjuvant chemotherapy post‐surgery usually does not increase postoperative risk [103, 104]. Therefore, for patients with early stage LC‐PTB, surgery remains the preferred treatment, with anti‐TB drug therapy initiated as soon as possible after surgery. All patients (except those with diabetes, immune system suppression, or defects) completed a six‐month course. These studies provide important references for the complex interactions between PTB and LC in terms of clinical management and surgical outcomes.

In the treatment of late‐stage LC‐PTB, the interaction between drugs needs to be considered. Some targeted drugs can affect anti‐TB drugs by regulating cytochrome P450 (CYP450)‐dependent metabolism and transport proteins, thereby altering their in vivo levels. Additionally, different types of traditional chemotherapy drugs can affect anti‐TB drugs via various pathways [105, 106, 107, 108, 109, 110] (Table 2). Studies have shown that some anti‐cancer platinum compounds interact with hepatic microsomal CYP450, potentially affecting the metabolism of other drugs, such as rifampin [111]. Paclitaxel is affected by anti‐TB drugs, with rifampin inducing CYP3A4 and P‐glycoprotein (P‐gp) expression, affecting paclitaxel metabolism and efficacy, and promoting P‐gp overexpression through the CK2‐HSP90β‐PXP‐MDR1 signaling pathway and 14–3‐3*σ* protein interaction with the pregnane X receptor, affecting paclitaxel efficacy [112, 113]. Therefore, interactions between anticancer drugs and anti‐TB drugs may lead to changes in drug concentrations, toxicity, and efficacy. In clinical practice, physicians need to consider these interactions when prescribing drugs and make appropriate adjustments based on the patient's specific situation.

**TABLE 2:** Anti‐cancer drugs interact with anti‐tuberculosis drugs in the treatment of TB‐LC.

Anti‐cancer drugs	Metabolism	Anti‐tuberculosis drugs	Target	Interaction potential	An‐titumor effect	Management
Gefitinib	CYP3A4	Rifampin	EGFR	Inducer	AUC↓83%	With rifampin, up Gefitinib to 500 mg/day
Erlotinib	CYP3A4	Rifampin	EGFR	Inducer	Median AUC↓69%	Increase Erlotinib by 50 mg biweekly to a 450 mg max if tolerated
Osimertinib	CYP3A4	Rifampin	EGFR	Inducer	AUC↓78%	Increased the dose of Osimertinib to 160 mg qd when the two drugs were used together
Icotinib	CYP3A4	Rifampin	EGFR	Inducer	AUC↓45%	Avoid coadministration of rifampin
Alectinib	CYP3A4	Rifampin	ALK	Inducer	AUC0–∞ 73.2% ↓;M4 AUC0‐∞ 79% ↑; Sum alectinib and M4 AUC0–∞ 18.4% ↓	Be careful when combining alectinib with rifampin
Ceritinib	CYP3A4	Rifampin	ALK	Inducer	Single dose AUC0–∞ 70% ↓; Steady‐state AUC 67% ↓	Concomitant use of Ceritinib and rifampicin was avoided
Cobimetinib	CYP3A4	Rifampin	MEK	Inducer	AUC 83% ↓	Avoid using cobimetinib with rifampin together.
Dabrafenib	CYP3A4	Rifampin	BRAF	Inducer	AUC 34% ↓;Desmethyl dabrafenib AUC 30% ↓; Carboxy‐dabrafenib AUC 73% ↑	Simultaneous use is usually avoided
	Lenvatinib CYP3A4	Rifampin	VEGFR	Inducer	Single dose AUC0–∞ 30.6% ↑;Multiple doses AUC0–∞ 18.2% ↓	Rifampin doesn't markedly affect lenvatinib PK
Sunitinib	CYP3A4	Rifampin	VEGFR	Inducer	Sum sunitinib and SU12662 AUC0–∞ 46% ↓	Increase sunitinib by 12.5 mg steps to 87.5 mg daily with rifampin
Palbociclib	CYP3A4	Rifampin	CDK4/6	Inducer	AUC0–∞ 85.2% ↓	Avoid using palbociclib and rifampin together
Olaparib	CYP3A4	Rifampin	PARP	Inducer	tablet AUC0–∞ 87% ↓; capsule AUC 71% ↓	Avoid coadministration of rifampin
Sonidegib	CYP3A4	Rifampin	Smooth‐ ened	Inducer	Cancerpatients:sonidegib 1 day, rifampin 14 days AUC0–24 h 66% ↓; sonidegib 120 days, rifampin 120 days AUC0‐24 h 88% ↓; sonidegib 133 days, rifampin 14 days AUC0–24 h 80% ↓	With rifampin, consider upping Sonidegib to 400–800 mg daily
Afatinib	P‐gp	Rifampin	TKI	Inducer	In healthy subjects, AUC0–∞↓34%, Cmax↓22%	Afatinib was increased by 10 mg, with rifampin
Paclitaxel	CYP3A4/P‐gp	Rifampin	—	Inducer	CR↑	Monitor for reduced paclitaxel effectiveness in combination therapy.

Abbreviations: AUC, area under the plasma concentration–time curve; bid, bis in die; Cmax, maximum plasma concentration; CR, clearance rate; PK, pharmacokinetics; qd, quaque die.

Common adverse reactions to anticancer drugs include hematotoxicity, skin damage, liver damage, and gastrointestinal reactions. Common adverse reactions to anti‐TB drugs include gastrointestinal reactions, liver damage, rashes, and allergies. Whether they can be used simultaneously and whether adverse reactions will increase with concurrent use are concerns in clinical treatment. A retrospective study [114] on chemotherapy of late‐stage LC‐PTB patients found that concurrent chemotherapy and anti‐TB treatment in 33 LC‐PTB patients was safe, and PTB did not increase the risk of disease progression and death in LC patients. A clinical study by Xie et al. [115] on 1448 lung adenocarcinoma patients found that lung adenocarcinoma patients with PTB receiving targeted drug treatment (EGFR‐TKI) had shorter overall survival than the simple LC group (*p* < 0.005), with low rates of grade 3/4 adverse reactions in both groups. A 2021 study [116] evaluating the clinical efficacy of anti‐PD‐(L)1 treatment in 98 patients with coexisting malignant tumors and different TB statuses found that the objective response rates for patients with active PTB, LTBI, and obsolete PTB receiving immunotherapy were all greater than 70% (*p* > 0.005). The progression‐free survival (PFS) of the three groups was 8, 6, and 6 months, respectively, with no statistical differences (*p* > 0.005). The overall adverse event rate of simultaneous anti‐TB therapy and immunotherapy was 73.3%, with a grade 3–5 adverse reaction rate of 13.3%. Current research data indicate that for patients with LC‐PTB, treatment should include concurrent anti‐TB and anticancer therapy. In combination with chemotherapy, targeted therapy, or immunotherapy, there was no significant increase in adverse reactions to LC progression or death risk, aiding physicians in formulating treatment plans to improve treatment outcomes and reduce overall patient risk.

**FIGURE 1:**
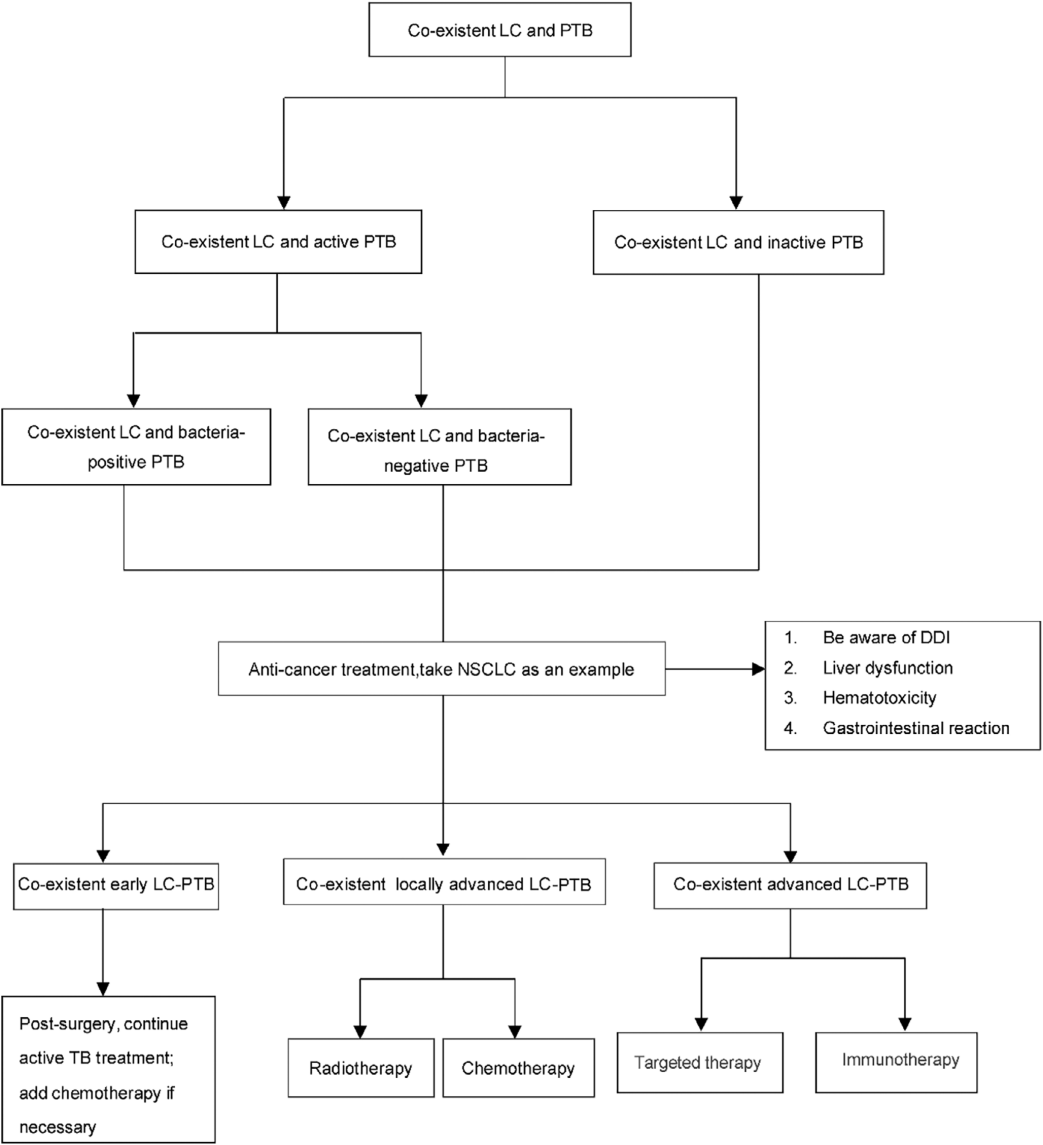
Management of lung cancer and tuberculosis co‐existence proposal algorithm.

In clinical treatment, the coexistence of TB and cancer presents significant challenges, including treatment plan selection, drug interactions, and prognosis. Patients suffering from both TB and lung cancer must be aware of the interaction between anti‐tuberculosis medications and EGFR‐TKIs, such as Osimertinib. It has been observed that tuberculosis infection may reduce the efficacy of TKI medications, complicating treatment outcomes [115]. Additionally, patient‐derived tumor‐like cell clusters (PTCs) have emerged as a promising platform for high‐throughput in vitro screening, which facilitates the modeling of complex drug combinations [117]. This approach holds substantial potential for enabling personalized treatment regimens tailored to individual patients' needs. Effective clinical management is crucial for improving treatment strategies and enhancing the quality of life for patients with both TB and cancer. Figure 1 outlines a clinical approach to managing this condition.

## 5 Conclusions

There is a strong relationship between TB and cancer in clinical practice, particularly with TB acting as a potential risk factor for cancer development, while cancer itself also influences susceptibility to TB. To systematically analyze the co‐morbidity mechanisms of TB‐associated lung cancer, it is crucial to explore its key molecular networks through multi‐omics integration (including genomics, epigenomics, metabolomics, etc.). Previous studies have shown that TB infection synergistically promotes lung carcinogenesis through multiple pathways. Chronic inflammation and immune imbalance play a central role, with TB‐induced persistent inflammatory responses and the accumulation of reactive oxygen species (ROS) leading to DNA damage. Additionally, mycobacterial effector proteins and mutations in genes such as EGFR contribute to the cancerous process. Current research on the TB‐associated tumor microenvironment focuses on the PD‐1/PD‐L1 axis; however, the expression patterns of other immune checkpoints, such as TIM‐3, LAG‐3, and TIGIT, and their relationship with T‐cell depletion, remain underexplored. Epigenetic reprogramming further extends the mechanistic understanding, as TB may alter the malignant phenotype of lung cancer cells by regulating key cancer‐related genes (e.g., oncogene silencing) through DNA methylation or histone modifications [118]. Notably, the spatial correlation between tuberculous lung scarring and ipsilateral lung carcinogenesis suggests that structural damage to the local microenvironment (e.g., fibrosis, hypoxia, and extracellular matrix remodeling) provides important clues about the spatio‐temporal evolution of TB‐related lung cancer [119]. Moreover, TB infection enhances type I interferon (IFN‐I) signaling in lung lymphocytes, with overexpression correlating positively with disease severity [120]. Sustained activation of IFN‐I in mouse models has been shown to exacerbate lung tissue damage [121]. Recent studies have highlighted the central role of plasmacytoid dendritic cells (pDCs) as key mediators of immune interactions in TB‐lung cancer through IFN‐I secretion, modulation of T‐cell responses, and maintenance of immune homeostasis [122, 123, 124]. However, relevant studies on this topic remain scarce.

To address these unresolved questions, particularly the causality of the TB‐lung cancer (TB‐LC) relationship and the development of targeted interventions, several types of studies are necessary. Longitudinal cohort studies are crucial for better understanding the temporal relationship between TB infection and the onset of lung cancer. These studies should track individuals with TB over time to determine the impact of chronic inflammation, immune imbalance, and DNA damage on lung cancer development. Additionally, randomized controlled trials (RCTs) or intervention studies should evaluate the potential benefits of therapies targeting immune modulation or inhibiting chronic inflammation in patients with TB at risk of developing lung cancer. Multi‐omics approaches integrating genomic, epigenomic, and metabolomic data will also be essential to uncover the molecular networks driving the TB‐lung cancer interaction. Further studies on the role of immune checkpoints, including PD‐1/PD‐L1, TIM‐3, LAG‐3, and TIGIT, in the tumor microenvironment are needed to explore their potential as therapeutic targets. Finally, clinical trials assessing the safety and efficacy of combination therapies—such as anti‐TB drugs combined with EGFR‐TKIs—are necessary to optimize treatment regimens for co‐infected patients and address potential drug interactions.

Although existing studies have revealed an association between pulmonary tuberculosis and lung cancer, the causal relationship between them is still unclear. Future studies should focus on longitudinal cohort studies to more clearly reveal the direct impact of TB on lung cancer development. At the same time, based on the mechanisms revealed in the current study, the development of targeted intervention strategies will be an important direction for future research. However, it is critical that further experimental research be conducted to elucidate the underlying biological mechanisms by which tuberculosis contributes to cancer development. This deeper understanding will be essential in improving treatment outcomes for co‐infected patients.

## Author Contributions

**Ning Su, Jinxing Hu:** conceptualization. **Jialou Zhu, Ning Su:** methodology. **Wendi Zhou, Yalin Xie:** formal analysis and investigation. **Wendi Zhou, Hongxu Lu:** writing – original draft preparation. **Ning Su, Jizhen Liang:** writing – review and editing. **Ning Su, Jinxing Hu, Jizhen Liang:** funding acquisition. **Hongxu Lu, Jiamin Lin:** visualization. **Ning Su, Jinxing Hu:** supervision.

## Conflicts of Interest

The authors declare no conflicts of interest.

## Data Availability

Data sharing is not applicable to this article as no new data were created or analyzed in this study.

## References

[1] S. Bagcchi , “WHO'S Global Tuberculosis Report 2022,” Lancet Microbe 4 (2023): e20. 36521512 10.1016/S2666-5247(22)00359-7

[2] Y. Luo , S. Suliman , S. Asgari , et al., “Early Progression to Active Tuberculosis Is a Highly Heritable Trait Driven by 3q23 in Peruvians,” Nature Communications 10 (2019): 3765. 10.1038/s41467-019-11664-1 PMC6704092 31434886

[3] J. A. Seddon and H. S. Schaaf , “Drug‐Resistant Tuberculosis and Advances in the Treatment of Childhood Tuberculosis,” Pneumonia 8 (2016): 20. 28702299 10.1186/s41479-016-0019-5 PMC5471710

[4] G. M. Knight , C. F. McQuaid , P. J. Dodd , and R. M. G. J. Houben , “Global Burden of Latent Multidrug‐Resistant Tuberculosis: Trends and Estimates Based on Mathematical Modelling,” Lancet Infectious Diseases 19 (2019): 903–912. 31281059 10.1016/S1473-3099(19)30307-X PMC6656782

[5] B.‐Y. Li , W. P. Shi , C. M. Zhou , et al., “Rising Challenge of Multidrug‐Resistant Tuberculosis in China: A Predictive Study Using Markov Modeling,” Infectious Diseases of Poverty 9 (2020): 65. 32513262 10.1186/s40249-020-00682-7 PMC7281937

[6] J. G. Jang and J. H. Chung , “Diagnosis and Treatment of Multidrug‐Resistant Tuberculosis,” Yeungnam University Journal of Medicine 37 (2020): 277–285. 32883054 10.12701/yujm.2020.00626 PMC7606956

[7] H. Sung , J. Ferlay , R. L. Siegel , et al., “Global Cancer Statistics 2020: GLOBOCAN Estimates of Incidence and Mortality Worldwide for 36 Cancers in 185 Countries,” CA: A Cancer Journal for Clinicians 71 (2021): 209–249. 33538338 10.3322/caac.21660

[8] C. Xia , X. Dong , H. Li , et al., “Cancer Statistics in China and United States, 2022: Profiles, Trends, and Determinants,” Chinese Medical Journal 135 (2022): 584–590. 35143424 10.1097/CM9.0000000000002108 PMC8920425

[9] P. Khan , J. A. Siddiqui , I. Lakshmanan , et al., “RNA‐Based Therapies: A Cog in the Wheel of Lung Cancer Defense,” Molecular Cancer 20 (2021): 54. 33740988 10.1186/s12943-021-01338-2 PMC7977189

[10] R. L. Siegel , K. D. Miller , N. S. Wagle , and A. Jemal , “Cancer Statistics, 2023,” CA: A Cancer Journal for Clinicians 73 (2023): 17–48. 36633525 10.3322/caac.21763

[11] M. Elhadi , A. Khaled , and A. Msherghi , “Infectious Diseases as a Cause of Death Among Cancer Patients: A Trend Analysis and Population‐Based Study of Outcome in the United States Based on the Surveillance, Epidemiology, and End Results Database,” Infectious Agents and Cancer 16 (2021): 72. 34972537 10.1186/s13027-021-00413-z PMC8719405

[12] K. Xiong , W. Sun , Y. He , and L. Fan , “Advances in Molecular Mechanisms of Interaction Between Mycobacterium Tuberculosis and Lung Cancer: A Narrative Review,” Translational Lung Cancer Research 10 (2021): 4012–4026. 34858788 10.21037/tlcr-21-465 PMC8577982

[13] V. Cukic , “The Association Between Lung Carcinoma and Tuberculosis,” Medical Archives 71 (2017): 212–214. 28974836 10.5455/medarh.2017.71.212-214 PMC5585804

[14] C. Y. Leung , H. L. Huang , M. M. Rahman , et al., “Cancer Incidence Attributable to Tuberculosis in 2015: Global, Regional, and National Estimates,” BMC Cancer 20 (2020): 412. 32398031 10.1186/s12885-020-06891-5 PMC7218646

[15] G.‐L. Chen , L. Guo , S. Yang , and D.‐M. Ji , “Cancer Risk in Tuberculosis Patients in a High Endemic Area,” BMC Cancer 21 (2021): 679. 34107921 10.1186/s12885-021-08391-6 PMC8190842

[16] Y.‐H. Yu , C. C. Liao , W. H. Hsu , et al., “Increased Lung Cancer Risk Among Patients With Pulmonary Tuberculosis: A Population Cohort Study,” Journal of Thoracic Oncology 6 (2011): 32–37. 21150470 10.1097/JTO.0b013e3181fb4fcc

[17] H. Abdeahad , M. Salehi , A. Yaghoubi , A. H. Aalami , F. Aalami , and S. Soleimanpour , “Previous Pulmonary Tuberculosis Enhances the Risk of Lung Cancer: Systematic Reviews and Meta‐Analysis,” Infectious Diseases 54 (2022): 255–268. 34807803 10.1080/23744235.2021.2006772

[18] C.‐M. Oh , Y. H. Roh , D. Lim , et al., “Pulmonary Tuberculosis Is Associated With Elevated Risk of Lung Cancer in Korea: The Nationwide Cohort Study,” Journal of Cancer 11 (2020): 1899–1906. 32194800 10.7150/jca.37022 PMC7052874

[19] W. Zheng , W. J. Blot , M. L. Liao , et al., “Lung Cancer and Prior Tuberculosis Infection in Shanghai,” British Journal of Cancer 56 (1987): 501–504. 2825752 10.1038/bjc.1987.233 PMC2001820

[20] J. Cabrera‐Sanchez , V. Cuba , V. Vega , P. Van Der Stuyft , and L. Otero , “Lung Cancer Occurrence After an Episode of Tuberculosis: A Systematic Review and Meta‐Analysis,” European Respiratory Review 31 (2022): 220025. 35896272 10.1183/16000617.0025-2022 PMC9724897

[21] M. E. Heuvers , J. G. J. V. Aerts , J. P. Hegmans , et al., “History of Tuberculosis as an Independent Prognostic Factor for Lung Cancer Survival,” Lung Cancer 76 (2012): 452–456. 22226628 10.1016/j.lungcan.2011.12.008

[22] K.‐M. Liao , C.‐S. Lee , Y.‐C. Wu , C.‐C. Shu , and C.‐H. Ho , “Prior Treated Tuberculosis and Mortality Risk in Lung Cancer,” Frontiers in Medicine 10 (2023): 1121257. 37064038 10.3389/fmed.2023.1121257 PMC10090669

[23] C. Y. Wu , H. Y. Hu , C. Y. Pu , et al., “Aerodigestive Tract, Lung and Haematological Cancers Are Risk Factors for Tuberculosis: An 8‐Year Population‐Based Study,” International Journal of Tuberculosis and Lung Disease 15 (2011): 125–130. 21276308

[24] K.‐M. Liao , C. C. Shu , F. W. Liang , et al., “Risk Factors for Pulmonary Tuberculosis in Patients With Lung Cancer: A Retrospective Cohort Study,” Journal of Cancer 14 (2023): 657–664. 37057286 10.7150/jca.81616 PMC10088535

[25] C. C. Dobler , K. Cheung , J. Nguyen , and A. Martin , “Risk of Tuberculosis in Patients With Solid Cancers and Haematological Malignancies: A Systematic Review and Meta‐Analysis,” European Respiratory Journal 50 (2017): 1700157. 28838977 10.1183/13993003.00157-2017

[26] C.‐C. Shu , K.‐M. Liao , Y.‐C. Chen , J.‐J. Wang , and C.‐H. Ho , “The Burdens of Tuberculosis on Patients With Malignancy: Incidence, Mortality and Relapse,” Scientific Reports 9 (2019): 11901. 31417132 10.1038/s41598-019-48395-8 PMC6695428

[27] S.‐W. Lai , C.‐L. Lin , and K.‐F. Liao , “Head and Neck Cancer Associated With Increased Rate of Pulmonary Tuberculosis in a Population‐Based Cohort Study,” Medicine 96 (2017): e8366. 29069025 10.1097/MD.0000000000008366 PMC5671858

[28] J. Zaemes and C. Kim , “Immune Checkpoint Inhibitor Use and Tuberculosis: A Systematic Review of the Literature,” European Journal of Cancer 132 (2020): 168–175. 32375103 10.1016/j.ejca.2020.03.015

[29] S. J. An , Y.‐J. Kim , S.‐S. Han , and J. Heo , “Effects of Age on the Association Between Pulmonary Tuberculosis and Lung Cancer in a South Korean Cohort,” Journal of Thoracic Disease 12 (2020): 375–382. 32274103 10.21037/jtd.2020.01.38 PMC7139000

[30] Q. Yang , M. Liao , W. Wang , et al., “CD157 Confers Host Resistance to *Mycobacterium Tuberculosis* via TLR2‐CD157‐PKCzeta‐Induced Reactive Oxygen Species Production,” MBio 10, no. 4 (2019): e01949‐19, 10.1128/mBio.01949-19. 31455656 PMC6712401

[31] S. Kwiatkowska , U. Szkudlarek , M. Łuczyńska , D. Nowak , and M. Zięba , “Elevated Exhalation of Hydrogen Peroxide and Circulating IL‐18 in Patients With Pulmonary Tuberculosis,” Respiratory Medicine 101 (2007): 574–580. 16890418 10.1016/j.rmed.2006.06.015

[32] X. Li , M. Wang , S. Ming , et al., “TARM‐1 Is Critical for Macrophage Activation and Th1 Response in *Mycobacterium tuberculosis* Infection,” Journal of Immunology 207 (2021): 234–243. 10.4049/jimmunol.2001037 34183366

[33] D. M. Shin , C. S. Yang , J. Y. Lee , et al., “ *Mycobacterium Tuberculosis* Lipoprotein‐Induced Association of TLR2 With Protein Kinase C ζ in Lipid Rafts Contributes to Reactive Oxygen Species‐Dependent Inflammatory Signalling in Macrophages,” Cellular Microbiology 10, no. 9 (2008): 1893–1905, 10.1111/j.1462-5822.2008.01179.x. 18503635 PMC2785852

[34] L. M. Ellzey , K. L. Patrick , and R. O. Watson , “Mitochondrial Reactive Oxygen Species: Double Agents in *Mycobacterium tuberculosis* Infection,” Current Opinion in Immunology 84 (2023): 102366. 37453340 10.1016/j.coi.2023.102366 PMC10711692

[35] E. Panieri and M. M. Santoro , “ROS Homeostasis and Metabolism: A Dangerous Liason in Cancer Cells,” Cell Death & Disease 7 (2016): e2253. 27277675 10.1038/cddis.2016.105 PMC5143371

[36] J. N. Moloney and T. G. Cotter , “ROS Signalling in the Biology of Cancer,” Seminars in Cell & Developmental Biology 80 (2018): 50–64. 28587975 10.1016/j.semcdb.2017.05.023

[37] C. Ryu , H. Sun , M. Gulati , et al., “Extracellular Mitochondrial DNA Is Generated by Fibroblasts and Predicts Death in Idiopathic Pulmonary Fibrosis,” American Journal of Respiratory and Critical Care Medicine 196 (2017): 1571–1581. 28783377 10.1164/rccm.201612-2480OC PMC5754440

[38] Y. Qin , Y. Chen , J. Chen , K. Xu , F. Xu , and J. Shi , “The Relationship Between Previous Pulmonary Tuberculosis and Risk of Lung Cancer in the Future,” Infectious Agents and Cancer 17 (2022): 20. 35525982 10.1186/s13027-022-00434-2 PMC9078090

[39] S.‐J. Kim , P. Cheresh , R. Jablonski , D. Williams , and D. Kamp , “The Role of Mitochondrial DNA in Mediating Alveolar Epithelial Cell Apoptosis and Pulmonary Fibrosis,” IJMS 16 (2015): 21486–21519. 26370974 10.3390/ijms160921486 PMC4613264

[40] W. J. Boyle , T. Smeal , L. H. K. Defize , et al., “Activation of Protein Kinase C Decreases Phosphorylation of c‐Jun at Sites That Negatively Regulate Its DNA‐Binding Activity,” Cell 64 (1991): 573–584. 1846781 10.1016/0092-8674(91)90241-p

[41] C. Timblin , K. BeruBe , A. Churg , et al., “Ambient Particulate Matter Causes Activation of the c‐Jun Kinase/Stress‐activated Protein Kinase Cascade and DNA Synthesis in Lung Epithelial Cells,” Cancer Research 58, no. 20 (1998): 4543–4547. 9788597

[42] Y. Wook Chung , D. W. Jeong , J. Yun Won , E. J. Choi , Y. Hyun Choi , and I. Young Kim , “H2O2‐Induced AP‐1 Activation and Its Effect on p21WAF1/CIP1‐Mediated G2/M Arrest in a p53‐Deficient Human Lung Cancer Cell,” Biochemical and Biophysical Research Communications 293 (2002): 1248–1253. 12054510 10.1016/S0006-291X(02)00360-1

[43] S. Gupta , T. Hussain , and H. Mukhtar , “Molecular Pathway for (−)‐Epigallocatechin‐3‐Gallate‐Induced Cell Cycle Arrest and Apoptosis of Human Prostate Carcinoma Cells,” Archives of Biochemistry and Biophysics 410 (2003): 177–185. 12559991 10.1016/s0003-9861(02)00668-9

[44] L. M. Coussens and Z. Werb , “Inflammation and Cancer,” Nature 420 (2002): 860–867. 12490959 10.1038/nature01322 PMC2803035

[45] J. L. Flynn and J. Chan , “Immune Cell Interactions in Tuberculosis,” Cell 185 (2022): 4682–4702. 36493751 10.1016/j.cell.2022.10.025 PMC12162144

[46] M. Königshoff , “Lung Cancer in Pulmonary Fibrosis: Tales of Epithelial Cell Plasticity,” Respiration 81 (2011): 353–358. 21502777 10.1159/000326299

[47] R. K. Bobba , J. S. Holly , T. Loy , and M. C. Perry , “Scar Carcinoma of the Lung: A Historical Perspective,” Clinical Lung Cancer 12 (2011): 148–154. 21663856 10.1016/j.cllc.2011.03.011

[48] A. Nalbandian , B.‐S. Yan , A. Pichugin , R. T. Bronson , and I. Kramnik , “Lung Carcinogenesis Induced by Chronic Tuberculosis Infection: The Experimental Model and Genetic Control,” Oncogene 28 (2009): 1928–1938. 19330024 10.1038/onc.2009.32

[49] A. J. Ozga , M. T. Chow , and A. D. Luster , “Chemokines and the Immune Response to Cancer,” Immunity 54 (2021): 859–874. 33838745 10.1016/j.immuni.2021.01.012 PMC8434759

[50] M. Zhang , Y. Zhou , and Y. Zhang , “High Expression of TLR2 in the Serum of Patients With Tuberculosis and Lung Cancer, and Can Promote the Progression of Lung Cancer,” Mathematical Biosciences and Engineering 17 (2020): 1959–1972. 10.3934/mbe.2020104 32233518

[51] V. Bours , M. Bentires‐Alj , A. C. Hellin , et al., “Nuclear Factor‐κB, Cancer, and Apoptosis,” Biochemical Pharmacology 60 (2000): 1085–1089. 11007945 10.1016/s0006-2952(00)00391-9

[52] K. S. Alharbi , N. K. Fuloria , S. Fuloria , et al., “Nuclear Factor‐Kappa B and Its Role in Inflammatory Lung Disease,” Chemico‐Biological Interactions 345 (2021): 109568. 34181887 10.1016/j.cbi.2021.109568

[53] K. P. Pattanaik , G. Ganguli , S. K. Naik , and A. Sonawane , “ *Mycobacterium tuberculosis* EsxL Induces TNF‐α Secretion Through Activation of TLR2 Dependent MAPK and NF‐κB Pathways,” Molecular Immunology 130 (2021): 133–141. 33419561 10.1016/j.molimm.2020.11.020

[54] M. Asaad , M. Kaisar Ali , M. A. Abo‐kadoum , et al., “ *Mycobacterium tuberculosis* PPE10 (Rv0442c) Alters Host Cell Apoptosis and Cytokine Profile via Linear Ubiquitin Chain Assembly Complex HOIP‐NF‐κB Signaling Axis,” International Immunopharmacology 94 (2021): 107363. 33667868 10.1016/j.intimp.2020.107363

[55] J. Wang , P. Ge , L. Qiang , et al., “The Mycobacterial Phosphatase PtpA Regulates the Expression of Host Genes and Promotes Cell Proliferation,” Nature Communications 8 (2017): 244. 10.1038/s41467-017-00279-z PMC5557760 28811474

[56] L. Qiang , J. Wang , Y. Zhang , et al., “ *Mycobacterium tuberculosis* Mce2E Suppresses the Macrophage Innate Immune Response and Promotes Epithelial Cell Proliferation,” Cellular & Molecular Immunology 16 (2019): 380–391. 29572547 10.1038/s41423-018-0016-0 PMC6461940

[57] M. P. Etna , E. Giacomini , M. Severa , and E. M. Coccia , “Pro‐ and Anti‐Inflammatory Cytokines in Tuberculosis: A Two‐Edged Sword in TB Pathogenesis,” Seminars in Immunology 26 (2014): 543–551. 25453229 10.1016/j.smim.2014.09.011

[58] G. J. Martínez‐Colón , “Prostaglandin E2 as a Regulator of Immunity to Pathogens,” Pharmacology and Therapeutics 185 (2018): 135–146, 10.1016/j.pharmthera.2017.12.008. 29274705 PMC5898978

[59] K. Pintha , W. Chaiwangyen , S. Yodkeeree , M. Suttajit , and P. Tantipaiboonwong , “Suppressive Effects of Rosmarinic Acid Rich Fraction From Perilla on Oxidative Stress, Inflammation and Metastasis Ability in A549 Cells Exposed to PM via C‐Jun, P‐65‐Nf‐Κb and Akt Signaling Pathways,” Biomolecules 11 (2021): 1090. 34439757 10.3390/biom11081090 PMC8392772

[60] E. Fosslien , “Biochemistry of Cyclooxygenase (COX)‐2 Inhibitors and Molecular Pathology of COX‐2 in Neoplasia,” Critical Reviews in Clinical Laboratory Sciences 37 (2000): 431–502. 11078056 10.1080/10408360091174286

[61] E. Fosslien , “Review: Molecular Pathology of Cyclooxygenase‐2 in Cancer‐Induced Angiogenesis,” Annals of Clinical and Laboratory Science 31 (2001): 325–348. 11688844

[62] D. Saka , M. Gökalp , B. Piyade , et al., “Mechanisms of T‐Cell Exhaustion in Pancreatic Cancer,” Cancers 12 (2020): 2274. 32823814 10.3390/cancers12082274 PMC7464444

[63] Z. Y. Chen , “Reversion of T‐Cell Exhaustion in Chronic Tuberculosis Infection by Immune Checkpoint Blockade,” Chinese Bulletin of Life Sciences 4 (2020): 349–358.

[64] E. A. Langan , V. Graetz , J. Allerheiligen , D. Zillikens , J. Rupp , and P. Terheyden , “Immune Checkpoint Inhibitors and Tuberculosis: An Old Disease in a New Context,” Lancet Oncology 21 (2020): e55–e65. 31908308 10.1016/S1470-2045(19)30674-6

[65] Y. Chen , S. Wu , G. Guo , et al., “Programmed Death (PD)‐1‐Deficient Mice Are Extremely Sensitive to Murine Hepatitis Virus Strain‐3 (MHV‐3) Infection,” PLoS Pathogens 7 (2011): e1001347. 21750671 10.1371/journal.ppat.1001347 PMC3131267

[66] M. E. Keir , M. J. Butte , G. J. Freeman , and A. H. Sharpe , “PD‐1 and Its Ligands in Tolerance and Immunity,” Annual Review of Immunology 26 (2008): 677–704. 10.1146/annurev.immunol.26.021607.090331 PMC10637733 18173375

[67] P. Jayaraman , I. Sada‐Ovalle , T. Nishimura , et al., “IL‐1β Promotes Antimicrobial Immunity in Macrophages by Regulating TNFR Signaling and Caspase‐3 Activation,” Journal of Immunology 190 (2013): 4196–4204. 10.4049/jimmunol.1202688 PMC3622150 23487424

[68] P. Jayaraman , M. K. Jacques , C. Zhu , et al., “TIM3 Mediates T Cell Exhaustion During *Mycobacterium tuberculosis* Infection,” PLoS Pathogens 12 (2016): e1005490. 26967901 10.1371/journal.ppat.1005490 PMC4788425

[69] X. Nan , C. Xie , X. Yu , and J. Liu , “EGFR TKI as First‐Line Treatment for Patients With Advanced EGFR Mutation‐Positive Non‐Small‐Cell Lung Cancer,” Oncotarget 8 (2017): 75712–75726. 29088904 10.18632/oncotarget.20095 PMC5650459

[70] H. Liu , B. Zhang , and Z. Sun , “Spectrum of EGFR Aberrations and Potential Clinical Implications: Insights From Integrative Pan‐Cancer Analysis,” Cancer Communications 40 (2020): 43–59. 32067422 10.1002/cac2.12005 PMC7163653

[71] Y. Shi , J. S. K. Au , S. Thongprasert , et al., “A Prospective, Molecular Epidemiology Study of EGFR Mutations in Asian Patients With Advanced Non‐Small‐Cell Lung Cancer of Adenocarcinoma Histology (PIONEER),” Journal of Thoracic Oncology 9 (2014): 154–162. 24419411 10.1097/JTO.0000000000000033 PMC4132036

[72] I. K. Hwang , S. S. Paik , and S. H. Lee , “Impact of Pulmonary Tuberculosis on the EGFR Mutational Status and Clinical Outcome in Patients With Lung Adenocarcinoma,” Cancer Research and Treatment 51 (2019): 158–168. 29621876 10.4143/crt.2018.084 PMC6333978

[73] W.‐L. Cheng , P. H. Feng , K. Y. Lee , et al., “The Role of EREG/EGFR Pathway in Tumor Progression,” International Journal of Molecular Sciences 22 (2021): 12828. 34884633 10.3390/ijms222312828 PMC8657471

[74] N. Sunaga , K. Kaira , H. Imai , et al., “Oncogenic KRAS‐Induced Epiregulin Overexpression Contributes to Aggressive Phenotype and Is a Promising Therapeutic Target in Non‐Small‐Cell Lung Cancer,” Oncogene 32 (2013): 4034–4042. 22964644 10.1038/onc.2012.402 PMC4451140

[75] S. C. Cowley , R. Babakaiff , and Y. Av‐Gay , “Expression and Localization of the *Mycobacterium tuberculosis* Protein Tyrosine Phosphatase PtpA,” Research in Microbiology 153 (2002): 233–241. 12066895 10.1016/s0923-2508(02)01309-8

[76] J. Wang , B. X. Li , P. P. Ge , et al., “ *Mycobacterium tuberculosis* Suppresses Innate Immunity by Coopting the Host Ubiquitin System,” Nature Immunology 16 (2015): 237–245. 25642820 10.1038/ni.3096

[77] J. Wang , J. L. L. Teng , D. Zhao , et al., “The Ubiquitin Ligase TRIM27 Functions as a Host Restriction Factor Antagonized by *Mycobacterium tuberculosis* PtpA During Mycobacterial Infection,” Scientific Reports 6 (2016): 34827. 27698396 10.1038/srep34827 PMC5048167

[78] B.‐J. Shen , W.‐C. Lo , and H.‐H. Lin , “Global Burden of Tuberculosis Attributable to Cancer in 2019: Global, Regional, and National Estimates,” Journal of Microbiology, Immunology and Infection 55 (2022): 266–272. 10.1016/j.jmii.2021.02.005 33789827

[79] C. Lin , H. He , H. Liu , et al., “Tumour‐Associated Macrophages‐Derived CXCL8 Determines Immune Evasion Through Autonomous PD‐L1 Expression in Gastric Cancer,” Gut 68 (2019): 1764–1773. 30661053 10.1136/gutjnl-2018-316324

[80] Y. Pu and Q. Ji , “Tumor‐Associated Macrophages Regulate PD‐1/PD‐L1 Immunosuppression,” Frontiers in Immunology 13 (2022): 874589. 35592338 10.3389/fimmu.2022.874589 PMC9110638

[81] K. J. Ishii , S. Koyama , A. Nakagawa , C. Coban , and S. Akira , “Host Innate Immune Receptors and Beyond: Making Sense of Microbial Infections,” Cell Host & Microbe 3 (2008): 352–363. 18541212 10.1016/j.chom.2008.05.003

[82] C. M. Balch , A. B. Tilden , P. A. Dougherty , G. A. Cloud , and T. Abo , “Depressed Levels of Granular Lymphocytes With Natural Killer (NK) Cell Function in 247 Cancer Patients,” Annals of Surgery 198 (1983): 192–199. 6870377 10.1097/00000658-198308000-00014 PMC1353079

[83] M. Ramos‐Casals , J. R. Brahmer , M. k. Callahan , et al., “Immune‐Related Adverse Events of Checkpoint Inhibitors,” Nature Reviews Disease Primers 6 (2020): 38. 10.1038/s41572-020-0160-6 PMC9728094 32382051

[84] K. Liu , D. Wang , C. Yao , et al., “Increased Tuberculosis Incidence due to Immunotherapy Based on PD‐1 and PD‐L1 Blockade: A Systematic Review and Meta‐Analysis,” Frontiers in Immunology 13 (2022): 727220. 35663958 10.3389/fimmu.2022.727220 PMC9162333

[85] C. L. Day , D. A. Abrahams , R. Bunjun , et al., “PD‐1 Expression on *Mycobacterium tuberculosis* ‐Specific CD4 T Cells Is Associated With Bacterial Load in Human Tuberculosis,” Frontiers in Immunology 9 (2018): 1995. 30233588 10.3389/fimmu.2018.01995 PMC6127207

[86] E. Lázár‐Molnár , B. Chen , K. A. Sweeney , et al., “Programmed Death‐1 (PD‐1)–deficient Mice Are Extraordinarily Sensitive to Tuberculosis,” Proceedings of the National Academy of Sciences 107 (2010): 13402–13407. 10.1073/pnas.1007394107 PMC2922129 20624978

[87] K. D. Kauffman , S. Sakai , N. E. Lora , et al., “PD‐1 Blockade Exacerbates *Mycobacterium tuberculosis* Infection in Rhesus Macaques,” Science Immunology 6 (2021): eabf3861. 33452107 10.1126/sciimmunol.abf3861 PMC8300572

[88] L. B. Tezera , M. K. Bielecka , P. Ogongo , et al., “Anti‐PD‐1 Immunotherapy Leads to Tuberculosis Reactivation via Dysregulation of TNF‐α,” eLife 9 (2020): e52668. 32091388 10.7554/eLife.52668 PMC7058383

[89] S. Rotstein , H. Blomgren , B. Petrini , J. Wasserman , and E. Baral , “Long Term Effects on the Immune System Following Local Radiation Therapy for Breast Cancer. I. Cellular Composition of the Peripheral Blood Lymphocyte Population,” International Journal of Radiation Oncology, Biology, Physics 11 (1985): 921–925. 3157666 10.1016/0360-3016(85)90114-2

[90] N. A. Lockney , M. Zhang , C. G. Morris , et al., “Radiation‐Induced Tumor Immunity in Patients With Non‐Small Cell Lung Cancer,” Thoracic Cancer 10 (2019): 1605–1611. 31228354 10.1111/1759-7714.13122 PMC6610279

[91] Y. Choi , J. M. Noh , S. H. Shin , et al., “The Incidence and Risk Factors of Chronic Pulmonary Infection After Radiotherapy in Patients With Lung Cancer,” Cancer Research and Treatment 55 (2023): 804–813, 10.4143/crt.2022.1305. 36596726 PMC10372583

[92] C. M. Tilsed , S. A. Fisher , A. K. Nowak , R. A. Lake , and W. J. Lesterhuis , “Cancer Chemotherapy: Insights Into Cellular and Tumor Microenvironmental Mechanisms of Action,” Frontiers in Oncology 12 (2022): 960317. 35965519 10.3389/fonc.2022.960317 PMC9372369

[93] M. C. Merlano , N. Denaro , D. Galizia , et al., “How Chemotherapy Affects the Tumor Immune Microenvironment: A Narrative Review,” Biomedicine 10 (2022): 1822. 10.3390/biomedicines10081822 PMC9405073 36009369

[94] M. A. Aldabbagh , A. Abughasham , G. Alansari , et al., “The Prevalence of *Mycobacterium Tuberculosis* Infection Among Cancer Patients Receiving Chemotherapy in a Tertiary Care Center,” Cureus 14 (2022): e32068, 10.7759/cureus.32068. 36600835 PMC9803363

[95] R. E. A. Jacobs , P. Gu , and A. Chachoua , “Reactivation of Pulmonary Tuberculosis During Cancer Treatment,” International Journal of Mycobacteriology 4 (2015): 337–340. 26964818 10.1016/j.ijmyco.2015.05.015

[96] A. A. Thai , B. J. Solomon , L. V. Sequist , J. F. Gainor , and R. S. Heist , “Lung Cancer,” Lancet 398 (2021): 535–554. 34273294 10.1016/S0140-6736(21)00312-3

[97] S. Vyse and P. H. Huang , “Targeting EGFR Exon 20 Insertion Mutations in Non‐Small Cell Lung Cancer,” Signal Transduction and Targeted Therapy 4 (2019): 5. 30854234 10.1038/s41392-019-0038-9 PMC6405763

[98] R. L. Ferris , H. J. Lenz , A. M. Trotta , et al., “Rationale for Combination of Therapeutic Antibodies Targeting Tumor Cells and Immune Checkpoint Receptors: Harnessing Innate and Adaptive Immunity Through IgG1 Isotype Immune Effector Stimulation,” Cancer Treatment Reviews 63 (2018): 48–60. 29223828 10.1016/j.ctrv.2017.11.008 PMC7505164

[99] C. Jin and B. Yang , “A Case of Delayed Diagnostic Pulmonary Tuberculosis During Targeted Therapy in an EGFR Mutant Non‐Small Cell Lung Cancer Patient,” Case Reports in Oncology 14 (2021): 659–663. 33976649 10.1159/000514050 PMC8077664

[100] H. Y. Lee , J. W. Kim , and C. D. Yeo , “A Case of Tuberculosis Reactivation Suspected of Cancer Progression During Oral Tyrosine Kinase Inhibitor Treatment in a Patient Diagnosed as Non‐Small Cell Lung Cancer,” Journal of Thoracic Disease 9 (2017): E709–E713. 28932591 10.21037/jtd.2017.07.31 PMC5594125

[101] J. Wang , L.‐. N. Lee , C.‐. J. YU , Y.‐. J. Chien , and P.‐. C. Yang , “Factors Influencing Time to Smear Conversion in Patients With Smear‐Positive Pulmonary Tuberculosis,” Respirology 14, no. 7 (2009): 1012–1019, 10.1111/j.1440-1843.2009.01598.x. 19659516

[102] H.‐Y. Liang , X. L. Li , X. S. Yu , et al., “Facts and Fiction of the Relationship Between Preexisting Tuberculosis and Lung Cancer Risk: A Systematic Review,” International Journal of Cancer 125 (2009): 2936–2944. 19521963 10.1002/ijc.24636

[103] A. Sharipov , M. Tillyashaykhov , O. Nematov , et al., “Lung Cancer and Lung Tuberculosis: Our Results of Treatment in the Combined Lung Disease,” in 8.1 Thoracic Surgery PA2497 (European Respiratory Society, 2016).

[104] S. Evman , V. Baysungur , L. Alpay , et al., “Management and Surgical Outcomes of Concurrent Tuberculosis and Lung Cancer,” Thoracic and Cardiovascular Surgeon 65 (2017): 542–545. 27111500 10.1055/s-0036-1583167

[105] Z.‐Y. Xu and J.‐L. Li , “Comparative Review of Drug‐Drug Interactions With Epidermal Growth Factor Receptor Tyrosine Kinase Inhibitors for the Treatment of Non‐Small‐Cell Lung Cancer,” Oncotargets and Therapy 12 (2019): 5467–5484. 31371986 10.2147/OTT.S194870 PMC6636179

[106] N. Meca , A. Manzaneque , G. Castells , et al., “4CPS‐185 Administration of Oral Anticancer Drugs for Patients With Swallowing Difficulties,” in Section 4: Clinical Pharmacy Services (British Medical Journal Publishing Group, 2020).

[107] L. Molenaar‐Kuijsten , D. E. M. Van Balen , J. H. Beijnen , N. Steeghs , and A. D. R. Huitema , “A Review of CYP3A Drug‐Drug Interaction Studies: Practical Guidelines for Patients Using Targeted Oral Anticancer Drugs,” Frontiers in Pharmacology 12 (2021): 670862. 34526892 10.3389/fphar.2021.670862 PMC8435708

[108] C. R. Kucharczuk , A. Ganetsky , and J. M. Vozniak , “Drug‐Drug Interactions, Safety, and Pharmacokinetics of EGFR Tyrosine Kinase Inhibitors for the Treatment of Non–Small Cell Lung Cancer,” Journal of the Advanced Practitioner in Oncology 9 (2018): 189. 30588353 PMC6302998

[109] S. Wind , T. Giessmann , A. Jungnik , et al., “Pharmacokinetic Drug Interactions of Afatinib With Rifampicin and Ritonavir,” Clinical Drug Investigation 34, no. 3 (2014): 173–182, 10.1007/s40261-013-0161-2. 24399452

[110] K. Vishwanathan , P. A. Dickinson , K. So , et al., “The Effect of Itraconazole and Rifampicin on the Pharmacokinetics of Osimertinib,” British Journal of Clinical Pharmacology 84 (2018): 1156–1169. 29381826 10.1111/bcp.13534 PMC5980546

[111] V. Mašek , E. Anzenbacherová , M. Machová , V. Brabec , and P. Anzenbacher , “Interaction of Antitumor Platinum Complexes With Human Liver Microsomal Cytochromes P450,” Anti‐Cancer Drugs 20 (2009): 305–311. 19378397 10.1097/cad.0b013e328323a7a8

[112] S. W. Kim , M. Hasanuzzaman , M. Cho , et al., “Role of 14‐3‐3 Sigma in Over‐Expression of P‐Gp by Rifampin and Paclitaxel Stimulation Through Interaction With PXR,” Cellular Signalling 31 (2017): 124–134. 28077325 10.1016/j.cellsig.2017.01.001

[113] J. Chen and K. Raymond , “Roles of Rifampicin in Drug‐Drug Interactions: Underlying Molecular Mechanisms Involving the Nuclear Pregnane X Receptor,” Annals of Clinical Microbiology and Antimicrobials 5 (2006): 3. 16480505 10.1186/1476-0711-5-3 PMC1395332

[114] M.‐F. Ye , S. Su , Z. H. Huang , et al., “Efficacy and Safety of Concurrent Anti‐Tuberculosis Treatment and Chemotherapy in Lung Cancer Patients With Co‐Existent Tuberculosis,” Annals of Translational Medicine 8 (2020): 1143. 33240992 10.21037/atm-20-5964 PMC7576042

[115] Y. Xie , N. Su , W. Zhou , et al., “Concomitant Pulmonary Tuberculosis Impair Survival in Advanced Epidermal Growth Factor Receptor (EGFR) Mutant Lung Adenocarcinoma Patients Receiving EGFR‐Tyrosine Kinase Inhibitor,” Cancer Management and Research 13 (2021): 7517–7526. 34621133 10.2147/CMAR.S326349 PMC8491869

[116] S. Su , M. F. Ye , X. T. Cai , et al., “Assessment of Anti‐PD‐(L)1 for Patients With Coexisting Malignant Tumor and Tuberculosis Classified by Active, Latent, and Obsolete Stage,” BMC Medicine 19 (2021): 322. 34923987 10.1186/s12916-021-02194-z PMC8686368

[117] S. Yin , R. Xi , A. Wu , et al., “Patient‐Derived Tumor‐Like Cell Clusters for Drug Testing in Cancer Therapy,” Science Translational Medicine 12 (2020): eaaz1723. 32581131 10.1126/scitranslmed.aaz1723

[118] J. Phelan , P. F. de Sessions , L. Tientcheu , et al., “Methylation in *Mycobacterium tuberculosis* Is Lineage Specific With Associated Mutations Present Globally,” Scientific Reports 8 (2018): 160. 29317751 10.1038/s41598-017-18188-y PMC5760664

[119] S. Brett , E. M. Irusen , and C. F. N. Koegelenberg , “Pulmonary Scarring and Its Relation to Primary Lung Cancer,” African Journal of Thoracic and Critical Care Medicine 26 (2020): 8, 10.7196/AJTCCM.2020.v26i1.050. PMC8203054 34240014

[120] S. Akter , K. S. Chauhan , M. D. Dunlap , et al., “ *Mycobacterium tuberculosis* Infection Drives a Type I IFN Signature in Lung Lymphocytes,” Cell Reports 39 (2022): 110983. 35732116 10.1016/j.celrep.2022.110983 PMC9616001

[121] L. Moreira‐Teixeira , P. J. Stimpson , E. Stavropoulos , et al., “Type I IFN Exacerbates Disease in Tuberculosis‐Susceptible Mice by Inducing Neutrophil‐Mediated Lung Inflammation and NETosis,” Nature Communications 11 (2020): 5566. 10.1038/s41467-020-19412-6 PMC7643080 33149141

[122] S. Alculumbre , S. Raieli , C. Hoffmann , R. Chelbi , F. X. Danlos , and V. Soumelis , “Plasmacytoid Pre‐Dendritic Cells (pDC): From Molecular Pathways to Function and Disease Association,” Seminars in Cell & Developmental Biology 86 (2019): 24–35. 29444460 10.1016/j.semcdb.2018.02.014

[123] A. Del Prete , V. Salvi , A. Soriani , et al., “Dendritic Cell Subsets in Cancer Immunity and Tumor Antigen Sensing,” Cellular & Molecular Immunology 20 (2023): 432–447. 36949244 10.1038/s41423-023-00990-6 PMC10203372

[124] K. A. Heldwein and M. J. Fenton , “The Role of Toll‐Like Receptors in Immunity Against Mycobacterial Infection,” Microbes and Infection 4 (2002): 937–944. 12106786 10.1016/s1286-4579(02)01611-8

